# Impact of ursodeoxycholic acid on circulating lipid concentrations: a systematic review and meta-analysis of randomized placebo-controlled trials

**DOI:** 10.1186/s12944-019-1041-4

**Published:** 2019-04-06

**Authors:** Luis E. Simental-Mendía, Mario Simental-Mendía, Adriana Sánchez-García, Maciej Banach, Maria-Corina Serban, Arrigo F. G. Cicero, Amirhossein Sahebkar

**Affiliations:** 1 Unidad de Investigación Biomédica, Delegación Durango, Instituto Mexicano del Seguro Social, Mexico, Mexico; 2 Department of Orthopedics and Traumatology, Hospital Universitario “Dr. José E. González”, Facultad de Medicina, Universidad Autónoma de Nuevo León, Monterrey, NL Mexico; 3 Endocrinology Division, Hospital Universitario “Dr. José E. González”, Facultad de Medicina, Universidad Autónoma de Nuevo León, Monterrey, NL Mexico; 4 Department of Hypertension, WAM University Hospital in Lodz, Medical University of Lodz, Zeromskiego 113, Lodz, Poland; 5 Polish Mother’s Memorial Hospital Research Institute (PMMHRI), Lodz, Poland; 6 Department of Functional Sciences, Discipline of Pathophysiology, Victor Babes University of Medicine and Pharmacy, Timisoara, Romania; 7 Medical and Surgical Sciences Department, University of Bologna, Bologna, Italy; 8 Neurogenic Inflammation Research Center, Mashhad University of Medical Sciences, Mashhad, Iran; 9 Biotechnology Research Center, Pharmaceutical Technology Institute, Mashhad University of Medical Sciences, Mashhad, Iran; 10 School of Pharmacy, Mashhad University of Medical Sciences, Mashhad, Iran; 11 Department of Medical Biotechnology, School of Medicine, Mashhad University of Medical Sciences, P.O. Box: 91779-48564, Mashhad, Iran

**Keywords:** Ursodeoxycholic acid, Lipid profile, Total cholesterol, Triglycerides, LDL, HDL, Meta-analysis

## Abstract

**Objective:**

The aim of this meta-analysis of randomized placebo-controlled trials was to examine whether ursodeoxycholic acid treatment is an effective lipid-lowering agent.

**Methods:**

PubMed-Medline, SCOPUS, Web of Science and Google Scholar databases were searched in order to find randomized controlled trials evaluating the effect of ursodeoxycholic acid on lipid profile. A random-effect model and the generic inverse variance weighting method were used for quantitative data synthesis. Sensitivity analysis was conducted using the leave-one-out method. A random-effects meta-regression model was performed to explore the association between potential confounders and the estimated effect size on plasma lipid concentrations.

**Results:**

Meta-analysis of 20 treatment arms revealed a significant reduction of total cholesterol following ursodeoxycholic acid treatment (WMD: − 13.85 mg/dL, 95% CI: -21.45, − 6.25, *p* < 0.001). Nonetheless, LDL-C (WMD: -6.66 mg/dL, 95% CI: -13.99, 0.67, *p* = 0.075), triglycerides (WMD: − 1.42 mg/dL, 95% CI: -7.51, 4.67, *p* = 0.648) and HDL-C (WMD: -0.18 mg/dL, 95% CI: -5.23, 4.87, *p* = 0.944) were not found to be significantly altered by ursodeoxycholic acid administration. In the subgroup of patients with primary biliary cirrhosis, ursodeoxycholic acid reduced total cholesterol (WMD: − 29.86 mg/dL, 95% CI: -47.39, − 12.33, *p* = 0.001) and LDL-C (WMD: -37.27 mg/dL, 95% CI: -54.16, − 20.38, *p* < 0.001) concentrations without affecting TG and HDL-C**.**

**Conclusion:**

This meta-analysis suggests that ursodeoxycholic acid therapy might be associated with significant total cholesterol lowering particularly in patients with primary biliary cirrhosis.

## Introduction

The global prevalence of hypercholesterolemia among adults is still increased [1]. Abnormal lipid levels, frequently accompanied by central obesity, high blood pressure and type 2 diabetes, have been clearly identified as a major risk factor for cardiovascular disease [2]. Moreover, the high prevalence of overweight and obesity have led to the increase in lipid disorders [3]. Given that pharmacological treatment may be insufficient to achieve the recommended goals for lipid concentrations, alternative lipid-lowering therapies are needed to reduce the risk of atherosclerotic cardiovascular disease [4–12].

Ursodeoxycholic acid is a primary bile acid formed in the human liver [13, 14]. This hydrophilic molecule has a low toxicity and is usually used at a pharmacological dose of 10–15 mg/kg/day [14, 15]. Ursodeoxycholic acid is widely prescribed in the treatment of several cholestatic liver diseases such as cholesterol-gallstone dissolution, primary biliary cirrhosis and cholestasis of pregnancy [16, 17]. Evidence suggests that the therapeutic effects of ursodeoxycholic acid are explained by an increased hydrophilicity index of the bile acid pool, stimulation of hepatocellular and ductular secretions, cytoprotection against bile acid and cytokine-induced injury, immunomodulation and anti-inflammatory effects [17]. Additionally, some clinical trials have observed a significant decrease in total cholesterol levels after ursodeoxycholic acid treatment [18–20]; however, other studies found no beneficial effect of this bile acid on lipid metabolism [21–23]. Thus, the lipid-lowering activity of ursodeoxycholic acid is currently uncertain and remains to be elucidated. Therefore, the present meta-analysis of randomized placebo-controlled trials aimed to examine whether ursodeoxycholic acid treatment is an effective lipid-lowering agent.

## Materials and methods

### Search strategy

This study was designed according to the guidelines of the 2009 preferred reporting items for systematic reviews and meta-analysis (PRISMA) statement [24]. In order to find randomized controlled trials evaluating the effect of ursodeoxycholic acid on lipid profile, PubMed-Medline, SCOPUS, Web of Science and Google Scholar databases were searched using the following search terms within titles and abstracts (also in combination with MESH terms): (ursodeoxycholic acid) AND (cholesterol OR “low-density lipoprotein” OR LDL OR LDL-C OR LDL-cholesterol OR “high-density lipoprotein” OR HDL-cholesterol OR HDL-C OR triglyceride OR hyperlipidemia OR hyperlipidemic OR dyslipidemia OR dyslipidemic OR lipid OR lipoprotein). The wild-card term “*” was used to increase the sensitivity of the search strategy. The search was limited to articles published in English language. The literature was searched from inception to June 06, 2018.

### Study selection

Original studies were included if they met the following inclusion criteria: (1) being a randomized placebo-controlled trial with either parallel or cross-over design, (2) evaluating the effect of ursodeoxycholic acid on plasma/serum concentrations of lipids, and, (3) presentation of sufficient information on lipid concentrations at baseline and at the end of follow-up in each group or providing the net change values. Exclusion criteria were: (1) non-interventional trials, (2) lack of a placebo group for ursodeoxycholic acid treatment, (3) observational studies with case-control, cross-sectional or cohort design, and (4) lack of sufficient information on baseline or follow-up (or net change) lipid concentrations.

### Data extraction

Eligible studies were reviewed and the following data were abstracted: 1) first author’s name; 2) year of publication; 3) study design; 4) number of participants in the intervention and placebo groups; 5) dose and duration of treatment with ursodeoxycholic acid; 6) age, gender and body mass index (BMI) of study participants; and 7) circulating concentrations of lipids.

### Quality assessment

A systematic assessment of bias in the included randomized placebo-controlled clinical trials was performed using the Cochrane criteria [25]. The items used for the assessment of each study were as follows: adequacy of random sequence generation, allocation concealment, blinding of participants, personnel, outcome assessment, not addressing dropouts (incomplete outcome data), selective outcome reporting, and other potential sources of bias. According to the recommendations of the Cochrane Handbook, a judgment of “yes” indicated low risk of bias, while “no” indicated high risk of bias. Labeling an item as “unclear” indicated an unclear or unknown risk of bias.

#### Quantitative data synthesis

Meta-analysis was conducted using Comprehensive Meta-Analysis (CMA) V2 software (Biostat, NJ) [26]. Effect size was calculated as: (measure at the end of follow-up in the treatment group − measure at baseline in the treatment group) − (measure at the end of follow-up in the control group − measure at baseline in the control group). A random-effect model (using DerSimonian-Laird method) and the generic inverse variance weighting method were used to compensate for the heterogeneity of studies in terms of study design, treatment duration, and the characteristics of populations being studied [27]. All units were collated as mg/dL. Standard deviations (SDs) of the mean difference were calculated using the following formula: SD = square root [(SD_pre-treatment_)^2^ + (SD_post-treatment_)^2^ – (2R × SD_pre-treatment_ × SD_post-treatment_)], assuming a correlation coefficient (R) = 0.5. Effect sizes were expressed as weighted mean difference (WMD) and 95% confidence interval (CI). Inter-study heterogeneity was quantitatively assessed using the *I*^*2*^ index. In order to evaluate the influence of each study on the overall effect size, a sensitivity analysis was conducted using the leave-one-out method (i.e., removing one study each time and repeating the analysis) [28–30].

## Meta-regression

As a potential confounder of treatment response, treatment duration was entered into a random-effects meta-regression model to explore their association with the estimated effect size on plasma lipid concentrations.

## Publication bias

Evaluation of funnel plot, Begg’s rank correlation and Egger’s weighted regression tests were employed to assess the presence of publication bias in the meta-analysis. When there was an evidence of funnel plot asymmetry, potentially missing studies were imputed using the “trim and fill” method [31].

## Results

### Flow of study selection

**Fig. 1:**
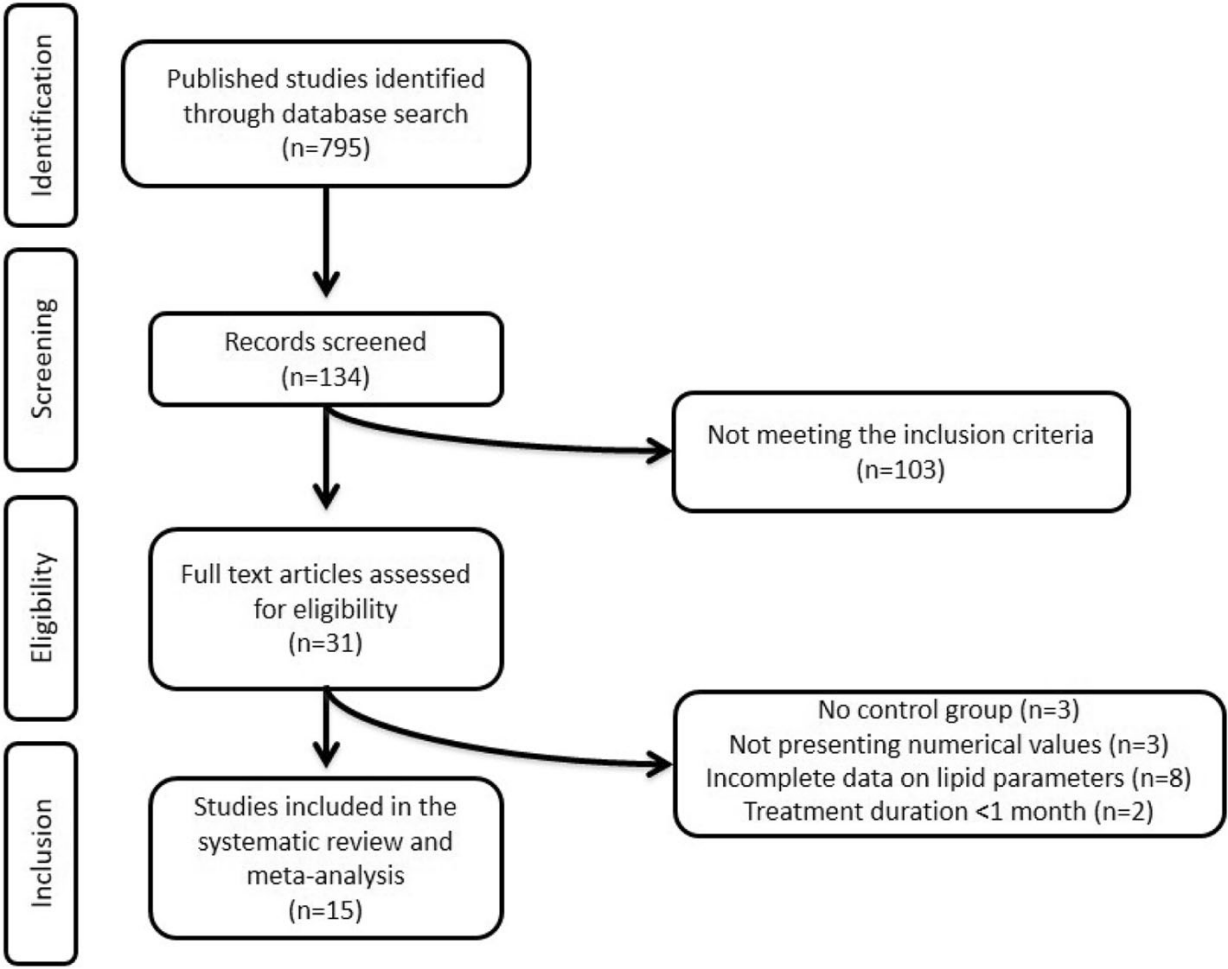
Flow chart of the number of studies identified and included in this meta-analysis

Our initial search identified 795 published trials. After screening of titles and abstracts, 661 studies were excluded. Of these, 103 studies excluded for not meeting the inclusion criteria. Subsequently, 31 full-text articles were carefully reviewed for eligibility and 16 clinical trials were excluded for having no control group (*n* = 3), not presenting numerical values (*n* = 3), incomplete data on lipid parameters (*n* = 8), and treatment duration < 1 month (*n* = 2). Finally, 15 studies were selected and included in the present meta-analysis. The detailed study selection process is presented in Fig. 1.

### Characteristics of included studies

Data were pooled from 15 randomized placebo-controlled trials comprising a total 1370 subjects, including 735 and 635 participants in the intervention and placebo arms, respectively. Included studies were published between 1977 and 2013. The clinical trials used different doses of ursodeoxycholic acid. The range of treatment duration was from 1 month [32, 33] to 2 years [18, 20, 23, 34–36]. Study design of included trials was parallel and cross-over. Selected studies enrolled subjects with primary biliary cirrhosis [18, 20, 21, 34–38], primary hypercholesterolemia [22], hypertriglyceridemia [32], gallstones [23, 37], nonalcoholic fatty liver disease (NAFLD) [19, 39], nonalcoholic steatohepatitis (NASH) [40], and healthy volunteers [33]. Characteristics of the included clinical trials are shown in Table 1.

**Table 1:** Demographic characteristics of the included studies

Author	Study design	Target Population	Treatment duration	n	Study groups	Age, years	Female (n, %)	BMI, (kg/m^2^)	Total cholesterol (mg/dl)	LDL cholesterol (mg/dl)	HDL cholesterol (mg/dl)	Triglycerides (mg/dl)
Balan et al. (1994) [18]	Randomized, double-blind, placebo-controlled	Primary biliary cirrhosis	2 years	89	Placebo	52 ± 9.4	77 (87)	ND	277.5 ± 106.0	ND	60.9 ± 20.3	117.2 ± 70.7
				88	UDCA 13–15 mg/kg/day	54 ± 9.3	80 (91)	ND	288.3 ± 121.7	ND	63.1 ± 23.6	102.0 ± 50.4
Battezzati et al. (1993) [21]	Randomized, double-blind, placebo-controlled	Primary biliary cirrhosis	6 months	44	UDCA 500 mg/day	54 ± 2	37 (84)	ND	263 ± 12	ND	78 ± 6	ND
				44	Placebo	55 ± 2	41 (93)	ND	266 ± 13	ND	61 ± 5	ND
Braga et al. (2009) [22]	Randomized, double-blind, placebo-controlled	Primary hypercholeste-rolemia	6 months	57	UDCA 13–15 mg/kg/day	ND	ND	ND	241.1 ± 30	160.2 ± 23	47.7 ± 12	166.0 ± 70
				68	Placebo	ND	ND	ND	244.7 ± 29	160.6 ± 19	48.0 ± 12	180.8 ± 96
Carulli et al. (1981) [32]	Randomized, double-blind, placebo-controlled	Hypertriglyce-ridemia	1 month	8	UDCA 600 mg/day	40.4^a^	1 (12)	ND	266^a^	ND	38^a^	405^a^
				8	Placebo	40.2^a^	2 (25)	ND	254^a^	ND	39^a^	249^a^
Fromm et al. (1983) [23]	Randomized, double-blind, placebo-controlled	Patients with gallstones	2 years	12	Placebo	55 ± 10.3	10 (82)	ND	201 ± 7.6	ND	ND	162 ± 30.8
				12	UDCA 400 mg/day	56 ± 16.9	8 (67)	ND	227 ± 16.5	ND	ND	162 ± 20.6
				12	UDCA 800 mg/day	55 ± 16.9	6 (50)	ND	248 ± 17.7	ND	ND	180 ± 32.4
Gianturco et al. (2013) [39]	Randomized, double-blind, placebo-controlled	NAFLD	1 year	53	ALA 400 mg/day + UDCA 300 mg/day	65 ± 5	23 (43)	30 ± 2.1	203 ± 8	133 ± 9	45 ± 5	123 ± 11
				54	ALA 400 mg/day	60 ± 4	25 (46)	29.5 ± 2	208 ± 9	133 ± 10	49 ± 6	128 ± 15
				46	UDCA 300 mg/day	62 ± 6	21 (45)	29.7 ± 1.6	209 ± 10	136 ± 11	47 ± 7	127 ± 11
				47	Placebo	61 ± 4	23 (48)	29.3 ± 1.3	207 ± 7	138 ± 12	43 ± 8	129 ± 9
Leuschner et al. (2010) [40]	Randomized, double-blind, placebo-controlled	NASH	18 months	95	UDCA 23–28 mg/kg/day	41.4 (18–71)^a^	32 (33)	ND	148 ± 102	ND	ND	208 ± 111
				91	Placebo	45.0 (18–73)^a^	28 (30)	ND	162 ± 94	ND	ND	202 ± 111
Lindenthal et al. (2002) [33]	Randomized, placebo-controlled, cross-over	Healthy volunteers	1 month	20	Overall	19–38^b^	5 (25)	ND				
				20	UDCA 750 mg/day				186 ± 24	ND	ND	ND
				20	Placebo				186 ± 19	ND	ND	ND
Méndez-Sánchez et al. (2004) [19]	Randomized, double-blind, placebo-controlled	NAFLD	6 weeks	14	UDCA 1200 mg/day	39.7 ± 8	14 (100)	34.2 ± 4.2	196.1 ± 36.7	ND	ND	ND
				13	Placebo	37.8 ± 8	13 (100)	33.3 ± 1.6	177.7 ± 29.3	ND	ND	ND
Miettinen et al. (1995) [20]	Randomized, double-blind, placebo-controlled	Primary biliary cirrhosis	2 years	23	UDCA 12–15 mg/kg/day	50 ± 9	18 (78)	24.1 ± 2.8	250 ± 122	138 ± 39	65 ± 19	ND
				22	Placebo	57 ± 14	21 (95)	24.8 ± 3.7	226 ± 76	131 ± 21	51 ± 26	ND
Nakagawa et al. (1977) [37]	Randomized, double-blind, placebo-controlled	Patients with gallstones	6 months	13	Placebo	ND	ND	ND	196 ± 30	ND	ND	120 ± 33
				16	UDCA 150 mg/day	ND	ND	ND	192 ± 41	ND	ND	110 ± 37
				15	UDCA 600 mg/day	ND	ND	ND	193 ± 29	ND	ND	143 ± 59
Parés et al. (2000) [34]	Randomized, double-blind, placebo-controlled	Primary biliary cirrhosis	2 years	99	UDCA 14–16 mg/kg/day	57.4 ± 8.9	92 (92)	ND	276 ± 89	ND	ND	ND
				93	Placebo	53.5 ± 9.6	87 (93)	ND	276 ± 86	ND	ND	ND
Poupon et al. (1990) [35]	Randomized, double-blind, placebo-controlled	Primary biliary cirrhosis	6 months	70	UDCA 13–15 mg/kg/day	55 ± 11	66 (94)	ND	282 ± 73	ND	ND	ND
				68	Placebo	58 ± 9	60 (89)	ND	266 ± 65	ND	ND	ND
Poupon et al. (1993) [38]	Randomized, double-blind, placebo-controlled	Primary biliary cirrhosis	2 years	17	UDCA 13–15 mg/kg/day	55 ± 12	ND	ND	289 ± 66	155 ± 52	44 ± 17	93 ± 32
				16	Placebo	58 ± 8	ND	ND	273 ± 36	134 ± 38	41 ± 14	102 ± 49
Vuoristo et al. (1995) [36]	Randomized, double-blind, placebo-controlled	Primary biliary cirrhosis	2 years	31	Placebo	57^a^	27 (87)	24^a^	278 ± 105	189 ± 84	61 ± 21	106 ± 48
				30	UDCA 12–15 mg/kg/day	52^a^	22 (73)	24^a^	251 ± 86	158 ± 64	58 ± 42	115 ± 49

Values are expressed as mean ± SD

*Abbreviations*: *ND* no data, *BMI* body mass index, *IQR* interquartile range

^a^Mean only

^b^Range

### Risk of bias assessment

According to the Cochrane criteria, most of included studies showed insufficient information about random sequence generation and one study had a high risk of bias [38]. With respect to allocation concealment, several trials exhibited limited information. Regarding blinding of participants, personnel and outcome assessors, several studies revealed lack of information and one trial presented high risk of bias [33]. Finally, all the evaluated trials had low risk of bias for incomplete outcome data and selective outcome reporting. Details for the risk of bias assessment is presented in Table 2.

**Table 2:** Quality of bias assessment of the included studies according to the Cochrane guidelines

Study	Sequence generation	Allocation concealment	Blinding of participants, personnel and outcome assessors	Incomplete outcome data	Selective outcome reporting	Other sources of bias
Balan et al. (1994) [18]	U	U	L	L	L	U
Battezzati et al. (1993) [21]	L	L	L	L	L	L
Braga et al. (2009) [22]	U	U	U	L	L	U
Carulli et al. (1981) [32]	U	U	U	L	L	U
Fromm et al. (1983) [23]	U	U	U	L	L	U
Gianturco et al. (2013) [39]	L	L	U	L	L	U
Leuschner et al. (2010) [40]	U	U	U	L	L	U
Lindenthal et al. (2002) [33]	U	U	H	L	L	U
Méndez-Sánchez et al. (2004) [19]	L	L	U	L	L	U
Miettinen et al. (1995) [20]	U	U	U	L	L	U
Nakagawa et al. (1977) [37]	U	L	L	L	L	U
Parés et al. (2000) [34]	U	L	L	L	L	U
Poupon et al. (1990) [35]	U	U	U	L	L	U
Poupon et al. (1993) [38]	H	U	U	L	L	U
Vuoristo et al. (1995) [36]	U	U	U	L	L	U

*L* low risk of bias, *H* high risk of bias, *U* unclear risk of bias

### Effect of ursodeoxycholic acid on lipids

**Fig. 2:**
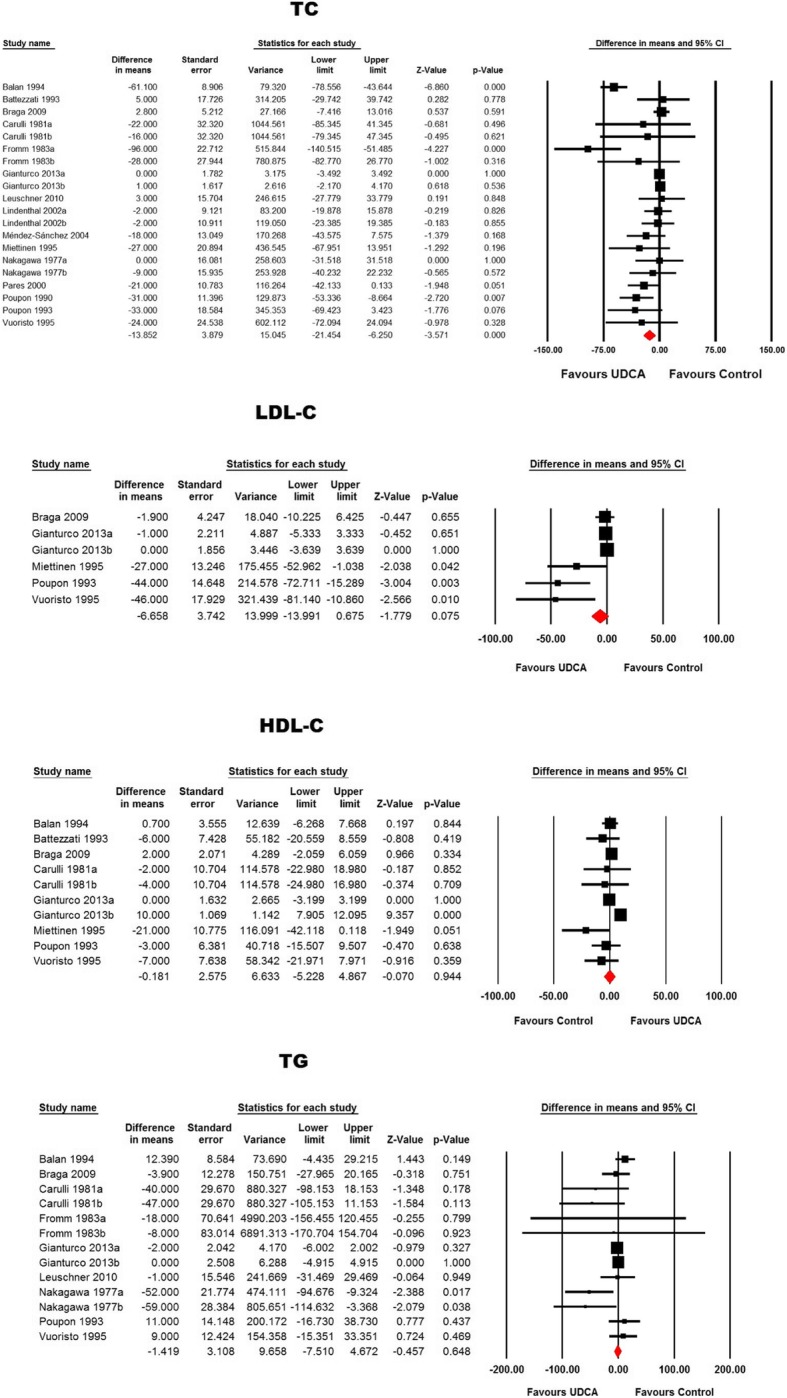
Forest plot displaying the weighted mean difference and 95% confidence intervals for the impact of treatment with UDCA on lipid indices

**Fig. 3:**
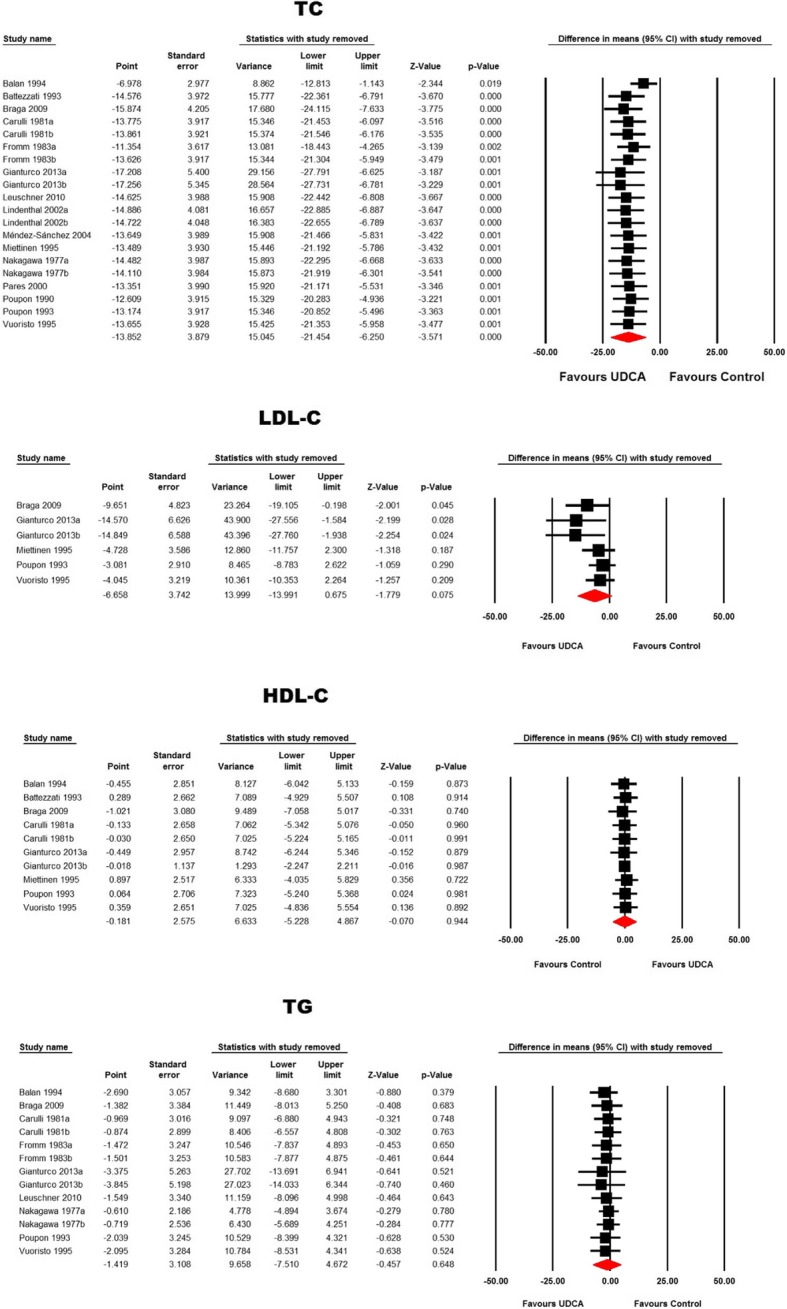
Leave-one-out sensitivity analysis for the meta-analysis of UDCA’s effects on plasma lipid indices

Meta-analysis of 20 treatment arms revealed a significant reduction of total cholesterol following ursodeoxycholic acid treatment (WMD: − 13.85 mg/dL, 95% CI: -21.45, − 6.25, *p* < 0.001). This effect size was robust in the sensitivity analysis **(**Figs. 2 and 3**).** Nonetheless, other lipid indices including LDL-C (WMD: -6.66 mg/dL, 95% CI: -13.99, 0.67, *p* = 0.075), TG (WMD: -1.42 mg/dL, 95% CI: -7.51, 4.67, *p* = 0.648) and HDL-C (WMD: -0.18 mg/dL, 95% CI: -5.23, 4.87, *p* = 0.944) were not found to be significantly altered by ursodeoxycholic acid administration (Figs. 2 and 3).

**Fig. 4:**
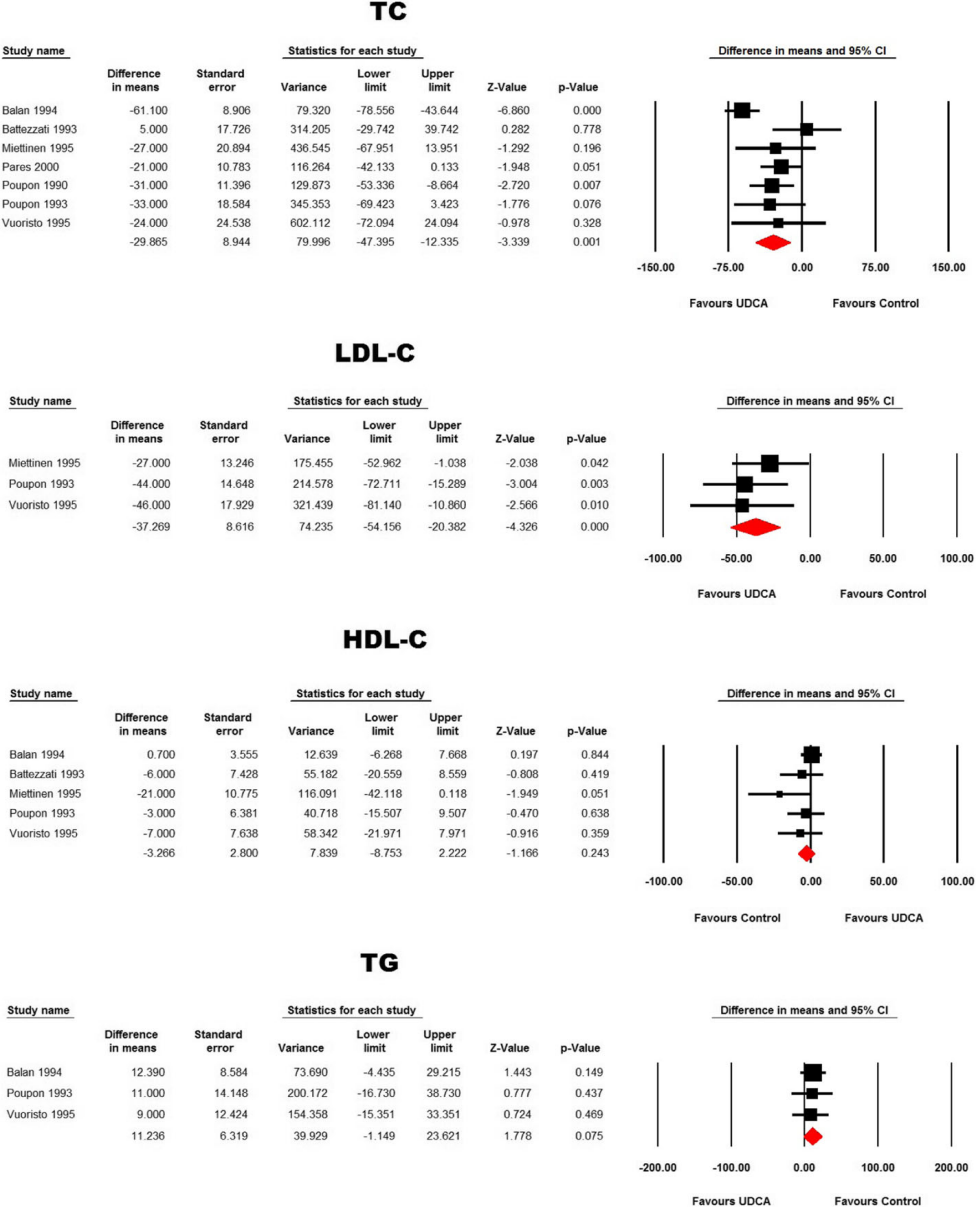
Forest plot displaying the weighted mean difference and 95% confidence intervals for the impact of treatment with UDCA on lipid indices in patients with primary biliary cirrhosis

In patients with primary biliary cirrhosis, ursodeoxycholic acid reduced total cholesterol (WMD: − 29.86 mg/dL, 95% CI: -47.39, − 12.33, *p* = 0.001) and LDL-C (WMD: -37.27 mg/dL, 95% CI: -54.16, − 20.38, *p* < 0.001) concentrations without affecting TG (WMD: 11.24 mg/dL, 95% CI: -1.15, 23.62, *p* = 0.075) and HDL-C (WMD: -3.27 mg/dL, 95% CI: -8.75, 2.22, *p* = 0.243) levels (Fig. 4)**.**

### Meta-regression

**Fig. 5:**
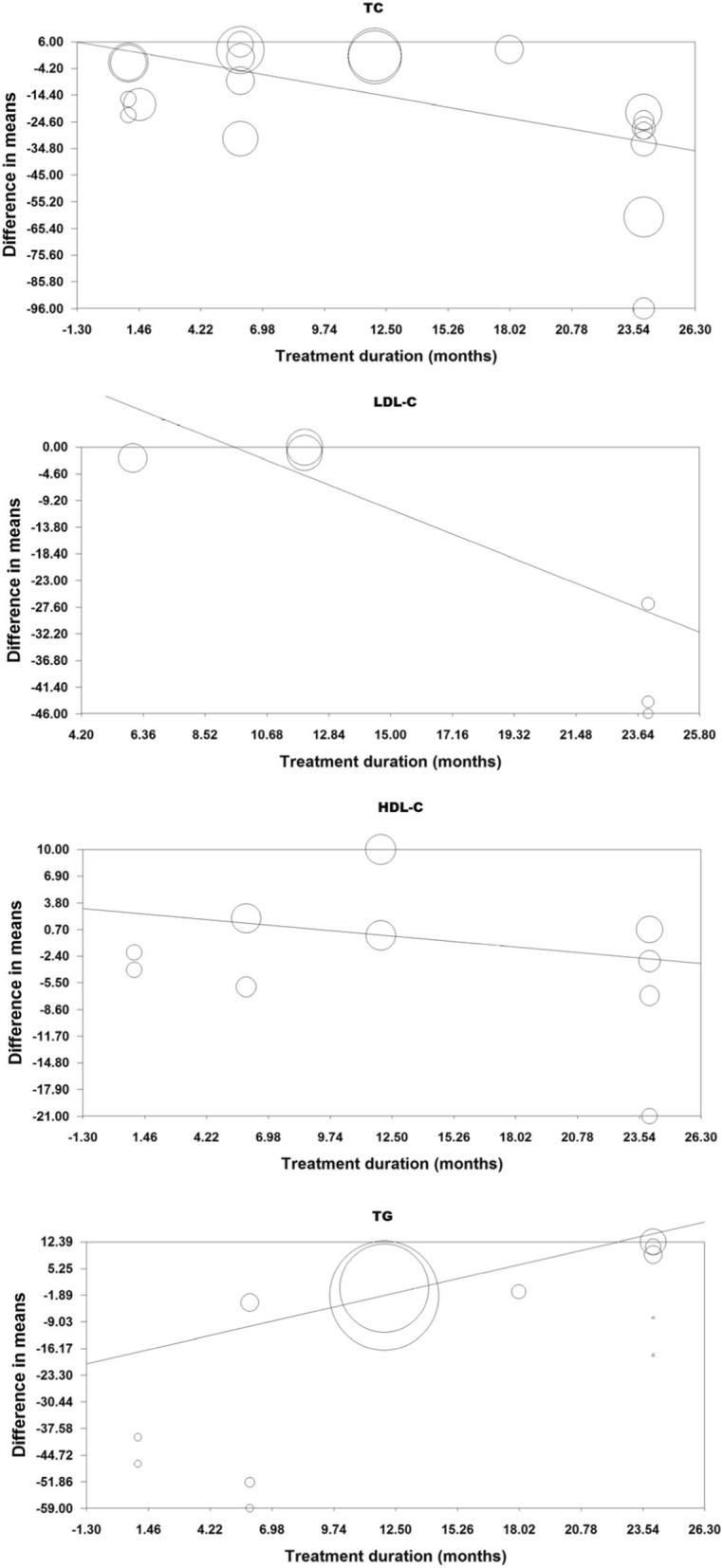
Meta-regression bubble plot of the association between mean changes in plasma lipids concentrations following UDCA supplementation with the duration of supplementation. The size of each circle is inversely proportional to the variance of change

Meta-regression analysis revealed that the effects of ursodeoxycholic acid on total cholesterol (slope: − 1.51; *p* < 0.001), LDL-C (slope: − 1.97; *p* = 0.001) and TG (slope: 1.38; *p* = 0.004) but not HDL-C (slope: − 0.23; *p* = 0.482) concentrations were associated with treatment duration (Fig. 5).

### Publication bias

**Fig. 6:**
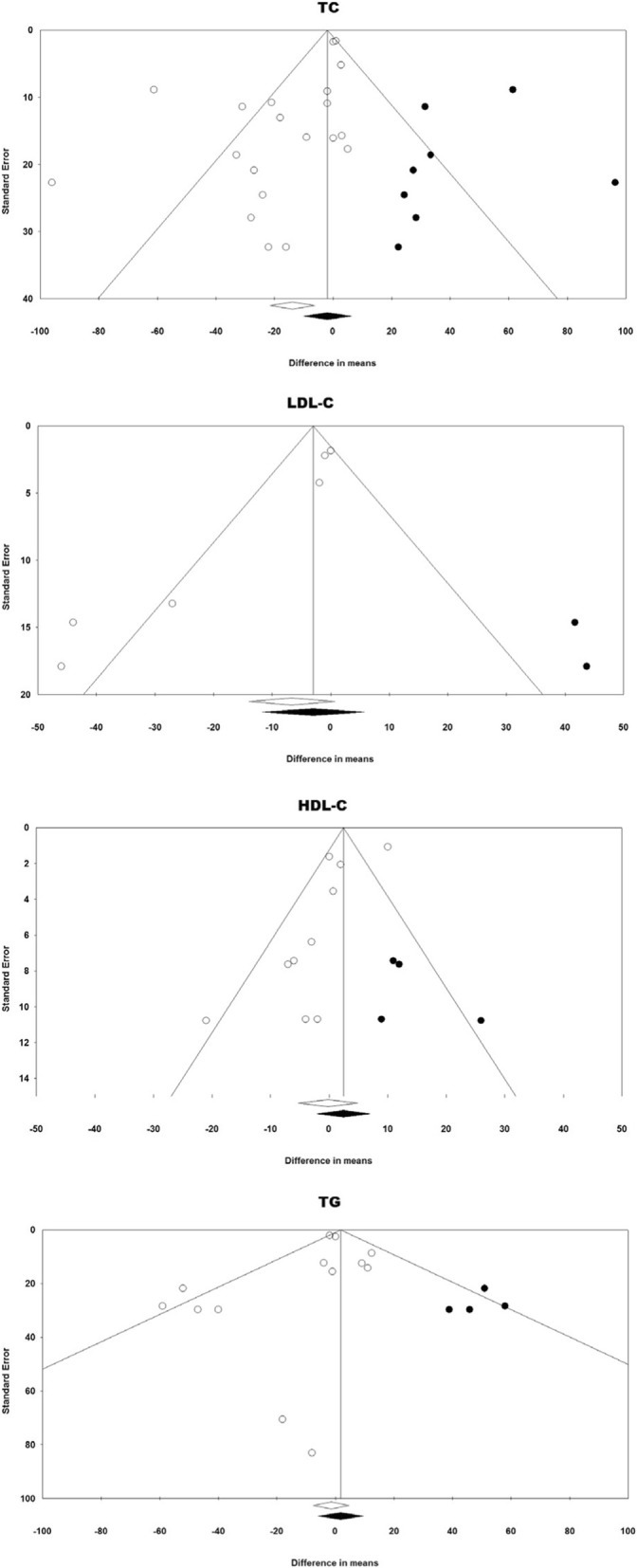
Funnel plot detailing publication bias in the studies reporting the impact of UDCA on lipid indices. Open circles represent observed published studies while closed circles represent imputed unpublished studies using trim and fill method

Publication bias assessment revealed asymmetric funnel plots and evidence suggestive of bias. This asymmetry was corrected by imputing potentially missing studies using “trim and fill” method (Fig. 6). Egger’s regression test suggested the presence of publication bias in the meta-analyses of total cholesterol (*p* = 0.008), LDL-C (*p* = 0.003) and HDL-C (*p* = 0.026). Begg’s rank correlation test suggested the presence of publication bias only in the meta-analysis of LDL-C (*p* = 0.024).

## Discussion

The present meta-analysis of randomized placebo-controlled trials examined whether ursodeoxycholic acid treatment might be an effective lipid-lowering agent. Indeed, this meta-analysis revealed a significant reduction in total cholesterol levels following ursodeoxycholic acid therapy (− 13.85 mg/dL), but the rest of parameters of lipid profile were not significantly changed.

In consistency with our findings, several clinical trials have found a significant reduction in total cholesterol concentrations after ursodeoxycholic acid administration [18, 19, 39, 40]; however, the potential mechanisms involved in the cholesterol-lowering effects of this bile acid have not been clarified. In this regard, it has been proposed that ursodeoxycholic acid may decrease the cholesterol biosynthesis by reducing the activity of hydroxymethylglutaryl-coenzime A reductase [41, 42]. Also, ursodeoxycholic acid decreases the dietary cholesterol absorption lowering serum cholesterol levels [43]. Additionally, it has been proven that the administration of ursodeoxycholic improves hepatic function through increasing the synthesis of bille acid, cholesterol and steatosis, and decreasing the activity of farnesoid X receptor (FXR) [44].

Experimental data suggested that ursodeoxycholic acid has also the ability to protect the cholangiocytes against hydrophobic bile acids by simultaneous decrease of the concentration of hydrophobic bile and reduction of the bile acid cytotoxicity [45]. Besides, it has been reported that this pharmacological agent increases hepatic LDL uptake through a direct interaction with the LDL receptor [46]. Furthermore, ursodeoxycholic acid was reported to be able to change the hydrophobicity index of the bile acid pool [47, 48]. Ursodeoxycholic acid may improve the cell resistance to reactive oxygen species, to decrease the permeability of the mitochondrial membrane and to inhibit release of hydrolytic enzymes from damaged hepatocytes [49, 50]. Moreover, some important genes involved in lipid uptake (*Cd36* and *Ldlr*) and hepatic lipid synthesis (PPARG, *Chrebp-a/−b*, *Acaca*, *Fasn*, *Me1*, and *Scd1*) seems to be modulated by ursodeoxycholic acid, as mecanisms of protection against hepatic fat accumulation [51]. Ursodeoxycholic acid may also influence the adipose tissue through increasing triglyceride levels, and increasing the esterification and desaturation of fatty acids [52].

Of particular interest is the clinically relevant decrease in TC and LDL-C specifically observed in primary biliary cirrhosis patients. This could be of particular interest given the increased coronary artery disease risk observed in patients affected by this condition [53].

There are some limitations of this meta-analysis that deserve to be mentioned. First, the lipid-lowering action of ursodeoxycholic acid was not the primary outcome in almost all selected studies; hence, further clinical trials are needed in order to corroborate the hypolipidemic effect of this acid bile as primary endpoint. Second, several studies included in this meta-analysis presented insufficient information with respect to the quality of bias assessment suggesting caution in the overall quality. Third, although the selected studies were heterogeneous in terms of target population and characteristics, we tried to minimize the inter-study heterogeneity using a random-effects model. Finally, most of the trials assessed were performed on small sample sizes resulting in a limited pooled population in the overall analysis.

## Conclusion

This meta-analysis suggests that ursodeoxycholic acid therapy might be associated with significant total cholesterol lowering. Nonetheless, these results could have been influenced by the variability, the sample size, and the quality of the studies included. Fuurther investigation is required to elucidate if observed lipid-lowering effects of ursodeoxycholic acid in patients with primary biliary cirrhosis can contribute to the prevention of cardiovascular events and whether there is any added value of using ursodeoxycholic acid as an adjunct or alternative to current or novel lipid-modifyinga gents [54, 55]
